# A qualitative exploration of internet forum discussions surrounding female sexual function for individuals with Sjögren’s syndrome

**DOI:** 10.1371/journal.pone.0291422

**Published:** 2023-09-08

**Authors:** Jemma L. McCready, Kristofor McCarty, Vincent Deary, Tracy L. Collins, Katie L. Hackett

**Affiliations:** 1 Department of Social Work, Education and Community Wellbeing, Faculty of Health and Life Sciences, Northumbria University, Newcastle upon Tyne, United Kingdom; 2 Department of Psychology, Faculty of Health & Life Sciences, Northumbria University, Newcastle upon Tyne, United Kingdom; 3 Newcastle upon Tyne Hospitals NHS Foundation Trust, Newcastle upon Tyne, United Kingdom; Chinese University of Hong Kong, HONG KONG

## Abstract

Sexual dysfunction is a common experience for women with the autoimmune rheumatic disease, Sjögren’s syndrome (SS); however, the lived experience of how the disease affects sexual functioning and the sexual environment remains unexplored. This qualitative study explores the conversations pertaining to female sexual function and the sexual environment that individuals with SS have on an internet forum. Qualitative data posted on one publicly accessible, worldwide, internet forum was extracted using an automated web scraping tool. A total of 247,694 posts across 23,382 threads were scraped from the forum in July 2019 and June 2022 (from the United Kingdom). A predetermined and theoretically informed keyword search strategy was used to screen the captured data for content relevant to the study aim. The dataset was cleaned to remove duplication and identifying information and screened for topic relevance. The Computer-Assisted Qualitative Data Analysis software tool, ATLAS.ti, was used to facilitate the data analysis process. Thematic analysis was conducted on 1443 female-oriented posts, and four key themes were identified: the symptoms of SS and their impact on the sexual environment; the emotional responses that are commonly evoked in response to sexual difficulties; the strategies that users have implemented to manage sexual problems; and the impact that a partner’s behavior may have on the sexual environment. Together these themes provide an insight into the nature of sexual difficulties for females with SS. Our findings provide novel insights to inform clinical discussions between practitioners and patients whilst further outlining the importance of undertaking qualitative research with this population.

## Introduction

Sjögren’s syndrome (SS) is an autoimmune rheumatic disease that attacks moisture-producing cells throughout the body [1]. Current estimates suggest that the disease affects between 400,000 and 3.1 million adults in the United States of America (USA) [2]. SS has a female preponderance (9:1) [3], and age at onset is typically between 40 to 60 years [4]. The disease can present independently, known as primary SS (pSS), or in conjunction with other rheumatic disorders such as rheumatoid arthritis (RA) and systemic lupus erythematosus (SLE) (secondary SS; sSS) [5]. The hallmark symptoms of SS are oral and ocular dryness. However, the systemic nature of the disease means that other organs and bodily systems can be affected (e.g., reproductive organs, nervous system), which can present as symptoms of vaginal dryness, joint and muscular pain, and fatigue [6].

Females diagnosed with SS commonly experience disruptions in sexual function and a decline in sexual quality of life. According to a recent systematic review and meta-analysis, approximately 74% of sexually active women with SS were found to have clinically significant sexual dysfunction, surpassing prevalence rates observed in other rheumatic conditions such as RA, SLE, and systemic sclerosis [7]. In particular, women with SS often encounter diminished sexual desire and arousal, heightened levels of discomfort during vaginal penetration, reduced sexual satisfaction, and more difficulty maintaining lubrication and reaching orgasm than healthy controls [8, 9]. These impairments in sexual functioning are attributed by women with SS to disease-related symptoms such as vaginal dryness, fatigue and joint or muscular pain [10]. Moreover, women with SS express dissatisfaction with both their sexual lives and the state of their relationship with their partners [9, 10]. In a cohort of 56 women with pSS, around a third reported that their relationship had become strained, and they perceived their partner to not be understanding of the alterations that had occurred in the sexual relationship [10].

Although sexual disturbances are common in SS, research suggests that a large proportion of women refrain from discussing sexual issues with their partners [10] and healthcare professionals (HCPs) [9, 11], citing reasons such as embarrassment or discomfort with raising the topic [9, 11]. Previous research has found that many patient groups turn to internet forums to discuss issues, seek support, and share experiences with other users experiencing similar problems [12]. Moreover, the ability to adopt an anonymous online persona has been shown to facilitate the discussion of sensitive or distressing topics, allowing users to feel more comfortable disclosing information about their experiences [13]. Studies that have qualitatively analyzed data from internet forums have found this to be an enriched and detailed source of data that generates similar themes to face-to-face interviews and adds additional depth to the topic under investigation [14].

Currently, there is a lack of research exploring the lived experience of sexual disruptions and the impacts on the sexual environment (e.g., the settings and behaviors surrounding sexual activity) with women with SS. Therefore, utilizing data from an internet forum will enable us to gather a ‘snapshot’ of the lived experience of sexual difficulties that individuals with SS face that they may not be comfortable discussing with healthcare professionals or in a research setting. Thus, this study aimed to qualitatively explore the conversations about sexual functioning that females with SS had on internet forums. We set out to explore the question: *what impact does SS have on sexual functioning and the sexual environment for females with SS*?

## Materials and methods

The Standards for Reporting Qualitative Research (SRQR) [15] were used to guide the reporting of this qualitative study.

### Ethical considerations

This research was granted ethical approval by the Northumbria University Ethics Committee (ref: 12220). User-generated data posted on a public internet forum is considered secondary data; as such, seeking informed consent for data usage from the individual is not typically required [16]. However, the British Psychological Society (BPS) urges researchers to consider their social responsibility and the impact the study could have on the social groups under investigation. For instance, announcing researchers’ presence on the internet forum could disrupt the social structures and cause users to disengage from what is an important place for them to access social support, thus causing harm to forum users [17]. Therefore, the research team decided that due to the nature of the internet forum and the sensitivity of the topic under investigation, it would be socially irresponsible and ethically harmful to seek informed consent from the forum users. To further minimize ethical harm in this study, the research team have taken the following decisions, as recommended by the BPS [16]. First, to respect the autonomy, privacy and dignity of the forum users, we decided only to include internet forums available in the public domain where data could be accessed or viewed without registering an account or obtaining a password. It is argued that by choosing publicly accessible forums, there is a degree of awareness by the users that the data they post can be observed by the wider public. Thus, it can be reasonably argued that not obtaining informed consent from forum users is justified [16, 18]. Moreover, to protect the anonymity of users, no personally identifiable information was scraped from the forums (e.g., usernames, IP addresses), and any identifiable information included within the content of posts was removed during data processing [19]. Furthermore, extracted quotations were checked before dissemination to ensure that internet searches could not trace the quote back to the originating forum archives and, subsequently, the individual [20, 21]. Additionally, the names and URLs of internet forums are not reported in dissemination outputs to further respect the forum user’s anonymity [21].

### Data sourcing

To identify eligible forums for inclusion in the study, a search was conducted on Google using the phrase ’Sjögren’s syndrome’ OR ’primary Sjögren’s syndrome’ AND ’internet forum’. The search identified 15 forums. However, only one forum was eligible for inclusion when assessed for eligibility (Table 1).

**Table 1:** Inclusion and exclusion criteria to assess forum eligibility.

Inclusion criteria	Exclusion criteria
• Forums dedicated solely to SS or pSS	• Forums that include the discussion of other health conditions
• Forums that are publicly available and do not require a password or user registration to access or view the forum	• Forums only accessible through the registration of an account
• Users of the forum need to be informed about the public nature of the forum	• Users are not informed that the site is accessible to the general public
• Research activities are permitted on the forum	• Research activities are prohibited
• The forum must contain a substantial number of postings	• A small number of postings

### Participant and forum characteristics

We cannot accurately report sociodemographic information for the participants due to their decision to refrain from sharing personal information (e.g., age, gender, diagnosis) on their user biographies (also known as bios). Moreover, the accuracy of any data shared on personal biographies cannot be presumed to be accurate. In accordance with other studies [22], a detailed summary of the forum will be provided to give the reader some understanding of the context in which the data was generated.

The forum used in this study was established in October 1999 and was designed to be a place where individuals with a diagnosis of SS or on the path to diagnosis can discuss issues, seek support, and share experiences with other users with SS. The forum was created by lay individuals diagnosed with SS rather than medical professionals or academic researchers. The forum’s content is user-generated and posted immediately without prior screening or approval by a moderator. Upon entering the forum, users are informed that the discussion boards are publicly accessible, and that content posted on the boards can be seen and used by members of the public. In addition, the forum’s guidelines inform the user that the forum does not prohibit research activities. Thus, the collection and analysis of the data in this study complied with the terms and conditions of the internet forum.

As of June 2022, the forum hosted approximately 2500 active members. The forum had no geographical restrictions for users, so it is assumed that the collected data may include users from various countries worldwide or predominantly from one particular country. However, the research team did not have access to this information. The forum contained eight discussion boards; six shared information about the site (e.g., community rules, moderator bios) and resources (e.g., academic articles), while the two remaining discussion boards were where users could discuss their experiences with SS with other users. Within a discussion board, users could either start a new discussion topic (known as a thread) or reply to existing threads (known as a post). At the time of the final data collection (June 2022), the entire forum contained 248,179 posts across 23,545 threads.

### Data acquisition

An automated web scraping tool (webscraper.io), accessed using the developer tools within Google Chrome, was used to scrape the data from the two discussion boards on the forum. Sitemaps were developed for each discussion board to allow the scraper to iterate through each thread and the pages contained within each thread (that show the post replies) dynamically (a process commonly called pagination). The scraper was programmed to extract the thread name and all posted content in each thread. No other data was scraped from the forum (e.g., profile information, IP addresses).

Data from the two main discussion boards were scraped at two-time points. The first data extraction phase began in the United Kingdom (UK) on 8^th^ July 2019 and captured content from 04/Apr/2008–30/Jul/2019. A total of 239,305 posts across 21,563 threads were extracted. An updated search was conducted on 27^th^ June 2022 and captured an additional 8389 posts across 1819 threads, which had been posted on the two discussion boards from 31/Jul/2019–27/Jun/2022 (Table 2). A total of 247,694 posts (99.8% capture rate) across 23,382 threads (99.3% capture rate) were scraped from the forum.

**Table 2:** Figures for threads and posts contained on each discussion board at the time of data extraction.

	Time-point 1: July 8^th^ 2019		Time-point 2: June 27^th^ 2022	
	Threads	Posts	Threads	Posts
Discussion board 1	19,233	212,062	20,848	219,122
Discussion board 2	2330	27,243	2534	28,572
Extraction total	21,563	239,305	23,382	247,694
Full forum	21,723	239,789	23,545	248,179

### Data processing

Data was initially stored in a database on a local machine (CouchDB). All posts were exported and parsed in JSON format and then written to CSV for analysis. Thread names and post content were written and organized into a single ‘dataframe’ object using the Pandas library in Python 3.7. A predetermined and theoretically informed keyword search string (Table 3) was applied to the dataset to query the thread names and post content relevant to the study’s aim.

**Table 3:** The search strategy used to screen the extracted data for relevant content.

(’sexual*’ OR ’intima*’ OR ’desire’ OR ’libido’ OR ’sex drive’ OR ’sex*’ OR ’sex* life’ OR ’arousal’ OR ’vagina*’ OR ’vaginal dryness’ OR ’orgasm’ OR ’lubrication’ OR ’vaginal atrophy’ OR ’dyspareunia’ OR ’sexual intercourse’ OR ’vulva*’ OR ’gyna*’ OR ’sexual partners’ OR ’partner’ OR ’pregnan*’)

The keyword search yielded a total of 3697 posts. Posts were hand-searched for duplication and relevance to the study topic. We identified 55 duplicated posts and 2121 posts that used the keywords in a context unrelated to the study aim (e.g., usernames, signoffs, shared informational resources). A total of 2176 posts were excluded at this stage. The narrative within the posts was checked to ensure a female-oriented discourse, and ambiguous or male-orientated discourses were excluded (n = 78). Included posts were manually cleaned to remove any identifying information (e.g., usernames) that users had included within their posts. A final total of 1443 female-orientated posts were uploaded into the Computer-Assisted Qualitative Data Analysis software tool, ATLAS.ti (Version 22) [23], for analysis.

### Theoretical considerations

This study intended to explore the experiences that women with SS encounter regarding their sexual function and their sexual lives and how this can be affected as a result of the illness. As there is an absence of qualitative research exploring the lived experience of the topic in SS, this study aims to take more of an exploratory stance, seeking to describe and understand rather than to explain the causes of the experience. As such, this research is informed by a phenomenological epistemological standpoint. This theoretical perspective aims to produce knowledge about subjective experiences, particularly the thoughts, feelings or perceptions that constitute the experience for an individual [24]. As this study was not concerned with the underlying causes or meanings beyond such experiences, a descriptive phenomenological epistemology was deemed more appropriate than an interpretative approach which reflects upon the experience within the broader social, cultural, and psychological context [25]. As this is an under-researched area, it was important for this study to identify common or shared experiences across a diversity of individuals, which would generate knowledge into how SS affects the sexual experiences of women with the condition. As a result, thematic analysis was chosen as the most appropriate method to facilitate this study’s aims and research question.

### Data analysis

Braun & Clarke’s thematic analysis [26, 27] was used to analyze the qualitative data contained in the posts. The first stage involved uploading the dataset to ATLAS.ti, where each post was categorized as a document and assigned a unique number. Each document was accessed in turn, and the post within was read numerous times until the researcher (JLM) was familiar with the dataset. An inductive, semantic approach was taken towards the coding of the posts. The dataset was coded iteratively, with codes being modified and added until all data was coded. The next stage involved sorting and organizing codes into meaningful themes. The preliminary themes were reviewed and revised through several team discussions (JLM, KLH, VD, TLC) and then refined, defined, and finalized. The final analysis contained four overarching themes.

## Results

The first theme provides an insight into the nature of sexual difficulties and the symptoms attributed by users to be responsible for these difficulties. The second theme encompasses the emotional responses commonly evoked in response to sexual difficulties. The third theme highlights the impact that a partner’s behavior may have on the sexual environment. The fourth theme outlines the treatment strategies that users have implemented to manage sexual problems. Together, these four themes provide an insight into the experiences, thoughts, feelings and behaviors of individuals with SS who visit an internet forum to discuss the impacts SS has on their sexual experiences and sexual relationship.

**“*Battling everything that goes with Sjögren’s sure puts the fire out*”—Symptoms of SS and their impact on the sexual environment**

### Decreased libido

Forum users provided detailed narratives of the impacts that disease-related symptoms had on their ability to engage in sexual activities. These typically referred to reductions in levels of sexual desire (libido), decreases in thinking about sex, and lack of willingness or interest to initiate sexual activity.

“*For me*, *it’s not so much a matter of lubrication or pain*, *rather than having lost desire and ability*.*”*

Many users reported that fatigue and pain were responsible for low libido as these affected their ability to feel “*in the mood*” and “*feel desirable*” and were said to hinder sexual “*spontaneity”* or *“playfulness”*. Some users also found it hard to differentiate which symptoms were responsible for sexual issues, for example:

“*I can’t say what affects me the most*, *the fatigue and by time kids go to bed I just want to lie on sofa like a dead duck*, *or pain in joints*, *or low mood*, *or lack of drive*, *or fear of pain puts me off*, *I can’t say*.*”*

### Reduced intimate non-sexual behaviors

Moreover, specific disease-related symptoms impact individuals’ ability to engage in sensual, intimate, and romantic behaviors. For example, allodynia, temperature dysregulation and widespread pain were reported by some users to impede their ability to cuddle, hold hands, be responsive to gentle touch, or share a bed with their partner. One user shared how their pain impacts their ability to cuddle:

“*It’s hard for me to even cuddle as my body hurts too much*. *If I lay on my side*, *my elbows and knees are in tremendous pain*.*”*

While another user described how allodynia affected touch responsivity from a partner and the perceived implications it had on the likelihood of sexual activity occurring:

“*He will reach for my hand and his touch hurts on top of the hurt*. *When my reflexes pull away*, *I’m sure he’s thinking well if I can’t hold her hand I’m not getting*… *[sex]*.*”*

The hallmark symptom of oral dryness was reported to have consequences on the comfortability of kissing, leading some users to avoid certain types of kissing, for example:

“*My partner noticed that I refused wet kisses during the last years*, *unconsciously I was only comfortable with kisses on the lips*. *I didn’t pay attention to this*. *But now that I know that I have Sjögren’s*, *I realize that of course it has relation with my mouth dryness*.*”*

### Vaginal dryness and resultant dyspareunia

Regarding sexual intercourse, users reported that vaginal dryness and reductions in vaginal lubrication made penetration painful and uncomfortable:

“*My vaginal dryness has gotten so bad that even with all the lubricating creams and gels out there*, *it is practically next to impossible to have sex*.*”*

The presence of dyspareunia caused some users to abstain from sexual intercourse for extended periods or, in some cases, entirely:

*“My hubby and I haven’t had sex in over a year and it does hurt*, *and I wish that we could*. *But it is so painful*, *and I have tried everything*.”

Users described how recurring episodes of dyspareunia caused them to experience a “*fear of pain*” during sexual activity, sometimes in the absence of any actual pain, which detracted from the enjoyment and pleasure of sexual intercourse. For example, one user wrote:

*“I hate that intimacy is something I now dread*, *and I try hard to hide that I really don’t want it anymore*. *Sometimes it actually does not hurt me*, *but I still can’t enjoy it for the fear of pain*.”

### Impaired body image

Moreover, several users disclosed that fatigue, pain, or dryness symptoms had negatively impacted their self-confidence and perceptions of their bodies. Some individuals reported that these psychological impacts also diminished their sexual drive and led to feelings of undesirability:

*“I see myself in the mirror and I hate what I see*. *I see in the mirror a dry*, *aching woman that hates herself*. *I just want to feel pretty again and to enjoy sex*.”

This theme highlights the impacts that certain disease-related symptoms (e.g., oral, and vaginal dryness, fatigue, and pain) can have on sexual functioning, specifically, reductions in levels of desire, difficulties engaging in romantic and intimate behaviors and a lack of enjoyment and satisfaction derived from sexual interactions.

**“*Guilt overwhelms me in this area*”–Emotional responses to sexual difficulties**

This theme and its subthemes captured how users discussed the emotional responses evoked when they experienced alterations in their sexual relationships. Feelings of guilt, grief and worry were the emotions most frequently reported.

### Feeling of guilt

Regarding guilt, users disclosed that they felt guilty for the impacts their illness had on their sexual relationship and their partner, for example:

*“Guilt overwhelms me in this area*, *and I have cried on my way to work many mornings thinking about how I couldn’t be intimate with my husband the night before because I was in just too much pain*, *or nauseous*, *or exhausted*.”

Users described how these emotions impacted them and how they tried to deal with the feelings. For example, some users reported that seeking emotional reassurance from their partners helped lessen the emotional burden, while others rationalized and assigned reasons to explain their feelings:

*“When I’m down and crying about the guilt I feel*, *he holds me in his arms and tells me how much I mean to him and how he couldn’t go on without me*.”

*“I too carry the guilt you all feel*, *but I try to remember*, *that I feel this way because I love him*.”

### A sense of grief

Another emotional response that was regularly discussed was grief. Users recounted how alterations in their sexual lives and sexual relationships amounted to feelings of loss and sacrifice, which induced an emotional yearning for a previous time when their sexual self and sexual relationships were unaltered. For example, two users wrote:

*“Sometimes I feel so cheated*, *why do we have to lose this too*, *on top of all the other sacrifices*?”

*“I feel like my intimacy has been obliterated by this awful disease*. *I would give anything to get it back again*.”

### Psychological distress

Other users expressed worry and anxiety when thinking about their ability to develop or maintain sexual relationships. For example, some users questioned their ability to develop a relationship in the future as they perceived oral and vaginal dryness to be a barrier to engaging in certain sexual behaviors (e.g., kissing, sexual intercourse):

*“I’ve also recently (last 3 months) started a relationship and I’m worried about the impact that this condition will have on our relationship*, *especially the intimacy side*.”

*“I am scared about the oral dryness*. *My mouth is unbearably dry*, *and I can’t ever imagine actually kissing a guy like this*. *I hate to think at age 27 I am never going to experience dating again and being loved*.”

Other users expressed emotional distress when thinking about maintaining sexual relationships with their partner and despaired over the prospect of relationship breakdown and separation. For example, one user shared:

*“What’s hard for me is that my hubby and I are in our early 30’s*, *supposed to be the prime of our sex life right but I feel like I’m cheating him in this area*. *I often feel like at times that I may drive him into the arms of another woman who can take care of his needs*.”

This theme sheds light on the emotional and psychological burden that alterations in sexual function due to SS can have on individuals and their perceived ability to develop or maintain relationships.

***“He stays mad because we aren’t intimate”*–The influence of the partner**

This theme and its subthemes encapsulate the other partner’s behaviors and attitudes when sexual difficulties are encountered.

### Positive response of supportiveness and understanding

Some users disclosed that their partners were supportive and understanding of the sexual difficulties that had arisen and would highlight how their partner would help them manage the illness’s impacts on the sexual relationship as well as within the sexual environment:

*“My husband is great about helping and understanding my limitations in the bedroom*, *and he’s pretty understanding if I need to stop*.”

*“I am very lucky in that my partner is extremely understanding of my dryness and will happily apply lube before*, *during and after sex*.”

Some users described how their partners would provide emotional support and reassurance when sexual difficulties arose. One user described their partner’s response when sexual problems were brought up in conversation:

*“I’ve told him about it and he’s very understanding*. *I’ve told him I’m worried about the sexual side of our relationship and he said that we are more than that and that we will cope with things*.”

Others revealed that their partner would help with household or childcare responsibilities to help minimize symptoms and conserve energy as a way to increase the likelihood of sexual activity occurring at a later time point:

*“If I think a certain day might be ’romantic’—& this is not more than 2 to 4 times a month—I curtail other energy-zapping activities (i*.*e*., *cleaning—which hubby picks up*, *best of both worlds*!*)*”

### Negative response with dissatisfied and altered sex life

In contrast, numerous users disclosed that their partners were neither supportive nor understanding of the situation and expressed dissatisfaction at the lack of sexual activity and the barriers in the sexual relationship. One user shared how their partner’s negative responses towards their altered sexual relationship further exacerbated the situation:

*“My husband is not supportive or helpful*, *or tolerant of the change in our sex life*. *Of course*, *the more he growls at me and tells me I’m having a pity party*, *the less I feel like even trying to have sex*.”

Some users reported feeling hostile and distant towards their partners and described their relationships as strained. Several individuals who reported this relationship dynamic also attributed this to be one of their reasons for sexual avoidance:

*“Well*, *I’m married but I have not had any sexual contact in nearly 7yrs*, *for many reasons*, *exhaustion*, *depression and the last couple of years a strained marriage as a result of my illnesses and depression*.”

Overall, this theme shows the influence that a partner’s behaviors and responses can have on the couple’s ability to adapt to any sexual difficulties that arise as a result of the disease. For example, our findings suggest that positive partner responses facilitate adjustment and that negative partner responses may exacerbate or create additional difficulties within the sexual relationship.

“*I have finally found a solution that works for me*”–The labor of love

This theme and its subthemes capture how users discussed and shared their strategies to reduce the impacts that vaginal and vulva dryness had on their sexual relationships.

### Management of vaginal dryness and irritation

These narratives also highlighted the behavioral, cognitive, and emotional labor that having SS had added to the sexual relationship, which resulted in the individual investing time, effort, and resources to find products and routines that offset the sexual disruptions caused by symptoms of SS:

*“It took a couple of years to figure out what worked for me*, *but I can tell you my husband is very pleased again*.”

### Preparation for sexual activity

The routines that individuals found helpful typically contained several products that were applied at different time points, although these routines differed depending upon the motives for use. For example, some individuals with SS had a routine for managing everyday vaginal dryness and genital discomfort (e.g., burning, itching, soreness):

*“I use Vagifem three times a week and then each day I use Replens*. *I’ve found that it soothes the burning and itching that I get throughout the day*.”

While others opted for routines that increased vaginal lubrication in preparation for sexual activity:

*“I will usually use one [a lubrication product] an hour before and then another right before*.”

### Post-intercourse care

Several individuals also outlined their after-sex routines, which mostly involved cleaning and applying products to their genitals to avoid a flare-up of symptoms (e.g., genital dryness and discomfort) in the hours following sexual activity:

*“After sex*, *I urinate and clean up thoroughly right away*, *no matter how tempting it is to just lay there*. *After that I smear Miconazole cream all over my vaginal parts*. *If I don’t do that clean up right away*, *I pay for it for a week with such irritation*.”

Some users discussed making vaginal pessaries, moisturizers, or soaps (typically containing coconut oil) to relieve SS-related dryness. One user described how they used coconut oil to manage vaginal dryness and the various ways in which they prepared the oil for use in certain situations:

*“If I need inner moisture*, *I take a small amount of the coconut oil and insert it*. *In the summer though it will be liquid*, *so I make the suppositories for inserting and keep them in the fridge*. *For yeast infections I make them with some powders probiotics in the oil to insert*.”

### Consultation with healthcare professionals

Many users reported finding these solutions themselves without guidance from HCPs. However, users who consulted HCPs reported being prescribed hormone-based products which typically contained estrogen:

*“My doctor has given me Vagifem*. *It is a small dose of estrogen that is inserted vaginally to give more lubrication (it helps a little)*.”

### Negative experiences managing vaginal dryness

However, not all users were able to find a solution or products that worked for them despite trying numerous suggestions:

*“I have tried every cream*, *lubricant*, *moisturizer on the market*, *and nothing*, *nothing at all helps give me any lubrication*.”

Additionally, some users described negative experiences with lubrication products, such as introducing such products into sexual activity, as well as experiencing unpleasant side effects:

*“We’ve tried all the over the counter lotions and it was awful*, *some of those products made my insides feel like a blowtorch had been used on me*.”

This theme sheds light on the additional work that individuals with SS must do to offset the disruptions caused by the disease to achieve a sense of normalcy in their sexual interactions.

## Discussion

This study explored the narratives surrounding female sexual functioning and the sexual environment that individuals with SS had on an internet forum. Our study found that lack of sex drive, vaginal dryness, fatigue, and pain were the symptoms commonly attributed as responsible for sexual difficulties. Our findings are in accordance with a study by Maddali Bongi and colleagues [10], who asked 38 women with pSS to report the symptoms that affect their sexual ability. Over half of the sample reported that vaginal dryness and lack of sex drive were responsible for alterations. They also found that around 20% of the sample reported that fatigue or pain (joint or muscular) impacted their sexual ability. However, the authors did not ask about the nature of these sexual changes, which is where this study may shed some light. Within the narratives, we found that specific symptoms of SS appeared to affect an individual’s ability to engage in intimate and romantic behavior, such as kissing, holding hands, and cuddling. Previous research has shown that engaging in intimate and romantic behaviors is important for provoking feelings of sexual desire and sexual willingness for some women [28]. Our findings suggest that individuals with SS may experience complications with initiating or being receptive to sexual cues due to disease-related symptoms.

Another symptom attributed to alterations in sexual function and the sexual environment was dyspareunia, which was perceived to be caused by vaginal dryness. This finding is not surprising as previous research has found that complaints of dyspareunia, specifically superficial dyspareunia (pain experienced in the vulval area or at the vaginal entrance) [29], are more common in women with SS than in healthy controls [8, 30]. However, in this study, details regarding the nature of the dyspareunia were sparse, and methodological limitations meant we were unable to probe further. Associations between the severity of vaginal dryness and experiences of dyspareunia have previously been reported [31, 32], which supports the subjective experiences reported by individuals in this study. Furthermore, our study highlights the psychological and behavioral alterations in response to dyspareunia. We found that for some users, recurring experiences of pain during sexual activity created a fear of pain, which, in some cases, led to disengagement or avoidance of sexual activity. For some users, this fear of pain persisted irrespective of actual pain experiences. Although these subjective experiences have not previously been reported in SS, they are similar to those reported by women experiencing dyspareunia due to other health conditions, such as endometriosis [33] and gynecological cancer [34]. Further research exploring the role of psychosocial factors in precipitating and perpetuating dyspareunia and sexual dysfunction in SS is needed, as this may provide a fruitful avenue to target intervention strategies.

Our study also offers insight into the emotional responses that females with SS experience when faced with alterations in sexual function and the sexual environment. Users discussed feeling a sense of grief at the loss or changes in their sexual relationships, guilt because of their illness’s impact on their partner and the sexual relationship and worries about developing and maintaining sexual relationships. Although not previously explored in women with SS, our findings are similar to previous qualitative studies exploring the lived experience of sexual alterations for women with reproductive cancers [34, 35]. In addition to our findings, these studies also provide a more in-depth exploration into the experiences of sexual loss, highlighting that alterations may also disrupt an individual’s sexual self-schema, sexual confidence and body image, all constructs that were not identified in this study. Further research is needed to explore this in more depth with women with SS.

Our findings also shed light on how a partner’s behaviors may play a role in adjusting to sexual difficulties. Within the narratives, we identified two contrasting experiences. The first was a group of users who reported that their partners were supportive and caring, understood the impacts of the illness on the sexual environment, provided emotional reassurance, and collaborated in finding solutions to sexual issues. The alternative narrative was centered on reports of partners being unsupportive and lacking compassion towards them and the sexual situation, as well as descriptions of a strained relationship. Although not specifically explored in SS, our findings are supported by previous research. For example, women who perceived their partners to exhibit negative behaviors in response to sexual difficulties (e.g., criticism, withdrawal from conversations, lack of support) also experienced more sexual dysfunction, poorer sexual adjustment, and less relationship satisfaction than those perceiving more positive partner behaviors [36]. Moreover, couples exhibiting poorer communication about sexual difficulties were more likely to experience sexual dysfunction and dissatisfaction with the relationship than those with more constructive forms of sexual communication [37, 38]. Supportive behaviors and constructive sexual communication between a couple may buffer against sexual dysfunction or improve sexual adjustment and are worthy of further investigation in this population group.

Within the symptom-management narratives, we found lubrication aids were one of the most discussed self-management options. This finding is not surprising given that a greater prevalence of usage of lubrication products was found in a population of women with pSS compared to healthy controls [11, 29]. Within our data, numerous users reported that lubrication products helped improve comfort during vaginal penetration and increased sexual satisfaction for both partners. This narrative could be supported by a study by Isik and colleagues [11], who found that women with pSS who used lubrication products reported less pain during vaginal penetration, greater sexual satisfaction, and significantly better sexual function than those not using lubrication aids. Although perceived to be helpful by forum users, research has found that repeated exposure to certain ingredients within lubrication products may cause damage to the vaginal epithelial cells [39, 40] and disrupt the vaginal microbiome [41] leading to symptoms of vaginal dryness and vaginal infections. As this population is already at risk of experiencing these symptoms [42], future research exploring the safety of long-term use of lubrication products for women with SS is imperative.

This study is also novel in that it provides insights into the cognitive and behavioral labor associated with managing the sexual disruptions caused by SS. The narratives suggest a journey of ‘trial and error’ in which a user becomes their own ‘case manager’ actively trying to find solutions that enable them to reclaim or maintain a sex-life and sexual identity. Users more successful in their endeavors engage in prosocial behavior, sharing details of management strategies, typically involving complex planning and preparation routines, usage of several products, and rigorous after-sex routines. Negative experiences resulting from ineffective products and unpleasant side effects lead to sexual avoidance and emotional distress for some women. Our data shows the cognitive and behavioral burden of dealing with sexual alterations and provides insights into the strategies deemed helpful by women with SS. Further exploration is required as these personal experiences may be valuable in developing more generalizable intervention strategies [43], which could help other women with SS self-manage sexual disruptions.

### Future research

The findings from this study have highlighted several areas that warrant further investigation. First and foremost, qualitative research using traditional methods should be conducted to gather a more holistic and contextualized understanding of the experience of altered sexuality in this population group. Moreover, researchers should explore the impacts that other factors, such as psychosocial adjustment to illness [44], coping strategies [45] and partners attitudes and behaviors [46] have on sexual functioning and sexual experiences for women with SS. Additionally, research should investigate the usage of lubrication products in this population to ensure that individuals are not causing themselves unnecessary harm. Findings from these research suggestions may identify potential areas for intervention which could ultimately help to support patients with SS to manage the disruptions that the illness has on their sexual functioning and sexual relationships.

### Implications for practice

The findings of this study emphasize the need for a comprehensive approach to address sexual difficulties for women with SS. Healthcare professionals should focus on symptom management, specifically, lack of sex drive, vaginal dryness, and dyspareunia—after ruling out any underlying causes. Pharmacological strategies to manage sexual alterations (e.g., hormones, lubrication aids) should be monitored and concerns should be addressed promptly to help avoid further sexual disruptions and minimize distress. The impacts sexual alterations have on the emotional wellbeing of the individual should be acknowledged, validated, and supported with personalized care where appropriate. Involving the partner in discussions around sexual wellbeing and encouraging supportive behaviors and effective communication is also an important strategy. By adopting a holistic, patient-centered approach, healthcare professionals can empower individuals to reclaim or maintain their sex lives and sexual identities. The importance of communication, education, and support in navigating the challenges of SS-related sexual disruptions is highlighted, ultimately aiming to improve the overall wellbeing and quality of life for individuals with SS.

### Limitations

This study has several limitations. Firstly, as this virtual ethnographic study observed naturally occurring discussions, we could not probe responses or ask follow-up questions to expand on the data. The data also lacked the contextual background typically present in qualitative studies. For example, we could not accurately report the number of users this dataset was drawn from, provide demographic details (e.g., age, disease duration, ethnicity), or even confirm health status and diagnosis of SS. Exploring this topic using traditional qualitative methods (e.g., one-to-one interviews) would facilitate greater transparency and control regarding participants’ characteristics. Moreover, the circumstances under which users typically engage in discussions on an internet forum must be considered. Individuals tend to seek support when experiencing significant emotional distress, complicated problems, or symptom flare-ups, and it may be that the data in this study captures the severer end of sexual disruptions. Additionally, the keywords used to search the captured dataset did not account for the casualness of language typically used on internet forums (e.g., unconventional language, spelling, and typographical symbols). As a result, we may have overlooked some relevant posts. It may be that future searches apply digital discourse frameworks to the search strings to account for variations in language, which would help to reduce selection bias.

## Conclusions

Overall, this study highlighted the physical, psychological, emotional, and social impacts of sexual disruptions, as well as the labor and complexity involved in finding and implementing self-management strategies to offset sexual disruptions caused by SS. In conclusion, collecting data from an internet forum proved to be a valuable method which has generated novel insights into the nature of sexual alterations for females with SS. Our findings can be used to inform clinical discussions between HCPs and patients, as well as provide a basis for future qualitative exploration.
